# Hyperglycemia in Cats Infected by SARS-CoV-2: Pancreatic Alterations and Potential Antiviral Therapeutics

**DOI:** 10.3390/microorganisms14091884

**Published:** 2026-08-25

**Authors:** Takashi Onodera, Sungwook Seo, Akikazu Sakudo, Antonio Toniolo

**Affiliations:** 1 Research Center for Food Safety, Graduate School of Agricultural and Life Sciences, The University of Tokyo, Bunkyo-ku 113-8657, Tokyo, Japan; 2 Nomura Research Institute, Chiyoda-ku 100-0004, Tokyo, Japan; seoandy1282@gmail.com; 3 Faculty of Veterinary Medicine, Okayama University of Science, Imabari 794-8555, Ehime, Japan; a-sakudo@ous.ac.jp; 4 Global Virus Network, University of Insubria, 21100 Varese, Italy; antonio.toniolo@gmail.com

**Keywords:** COVID-19, feline coronavirus, hyperglycemia, SARS-CoV-2

## Abstract

Cats represent a susceptible host and a possible translational model for investigating coronavirus pathogenesis and therapeutics. Recent immunohistochemical (IHC) and histopathological studies in both human and feline tissues have demonstrated SARS-CoV-2 nucleocapsid protein (NP) and spike protein expression within pancreatic islet cells, following a classic temporal infection course. Notably, IHC analysis also reveals NP expression within exocrine ductal epithelial cells. Given that ductal epithelium functions as an islet progenitor pool during tissue injury or metabolic stress, pancreotropic coronaviruses may gain access to the endocrine compartment by exploiting this intrinsic cellular differentiation pathway. Although the precise mechanisms governing intra-islet viral entry remain to be elucidated, this review highlights the capacity of SARS-CoV-2 to compromise both the exocrine (digestive) and endocrine functions of the pancreas. Finally, we evaluate the therapeutic potential of direct-acting antivirals—specifically RNA-dependent RNA polymerase (RdRp) and protease inhibitors—as monotherapies and in synergistic combination to limit pancreatic injury and mitigate the diabetogenic effects of coronaviruses across both acute infection and post-acute sequelae, such as human long-COVID syndrome.

## 1. Introduction

Insulin-dependent diabetes mellitus and pancreatitis impose substantial clinical, economic, and social burdens worldwide. A historical precedent for a viral etiology of insulin-dependent diabetes is well-established, particularly following enterovirus infections [1]. A classic example includes a case of diabetic ketoacidosis (DKA) in a child from whom Coxsackievirus B4 was isolated, a strain later proven to be directly diabetogenic in murine models [2]. Similar associations where Koch’s postulates appear applicable to human virus-induced diabetes have since been reported for Coxsackievirus B5 [3] and other enteroviruses [4]. A summary of viruses producing hyperglycemia and/or diabetes in animals is shown in Table 1.

More recently, attention has shifted to the pancreatic impact of the subfamily Orthocoronavirinae. Coronaviruses are large, enveloped, positive-sense RNA viruses (~85 nm in diameter) that infect a wide range of mammals and birds via respiratory droplets or fecal–oral transmission [12]. In humans, the beta-coronaviruses SARS-CoV, MERS-CoV, and SARS-CoV-2 are notorious for causing severe respiratory pathology, but increasing evidence suggests systemic complications. Concurrently, veterinary medicine has long recognized the impact of alpha-coronaviruses. In felids, two distinct biotypes exist based on pathogenicity [13]: Feline Enteric Coronavirus (FECV), which causes a benign gastrointestinal infection, and its mutated pathogenic variant, Feline Infectious Peritonitis Virus (FIPV), which induces a fatal systemic disease (FIP). Notably, viral pancreatitis has been documented in both cats and ferrets infected with FECV and FIPV [14,15].

This cross-species vulnerability is particularly evident with SARS-CoV-2, to which cats are extremely susceptible [16]. Recent studies demonstrate that cats infected with SARS-CoV-2 can exhibit severe hyperglycemia, overt diabetes, and metabolic crises like DKA or hyperosmolar states [17,18], findings mirrored in experimental feline models [19,20]. As shown in Table 1, there was no virus-induced hyperglycemia in feline models before 2022. Zhang et al. and Rochowski et al. developed experimental feline models of virus-induced hyperglycemia [19,20] during their animal experiments. However, the exact mechanisms driving coronavirus-mediated pancreatic damage and subsequent endocrine failure remain to be elucidated.

## 2. Virus-Induced Hyperglycemia in Humans and Animals

Though T1DM has historically been characterized as an acute juvenile-onset disease culminating in fatal ketoacidosis, contemporary research suggests a more nuanced, protracted progression toward complete insulin deficiency [21,22,23]. Current models favor an episodic decline, wherein successive waves of environmental or viral insults to beta-cells are interspersed with periods of partial clinical recovery [24]. This hypothesis is supported by the distinct seasonality of T1DM onset—which peaks during winter months in northern latitudes [25]—and may correlate with the circulation of specific “diabetogenic” enteroviruses, including Coxsackievirus B1, B2, B4, and B5, and Echovirus 9 and 11 [26]. Spatial and familial clustering within small populations further reinforces this infectious linkage [27,28].

Beyond epidemiological correlations, experimental models may provide evidence of viral tropism within the endocrine system. Murine inoculation with Coxsackievirus B4 triggers both acute and persistent pancreatic infections, alongside systemic manifestations such as myocarditis [28]. Similarly, human-isolated Reovirus types 1, 2, and 3—which typically exhibit gastrointestinal tropism—induce diabetes and hypothyroidism in neonatal mice following passage in primary beta-cell cultures. Ultrastructural analyses have localized crystalline arrays of Reovirus type 1 within the cytoplasm of pancreatic islet alpha-, beta-, and delta-cells [29], underscoring a broad affinity for diverse endocrine lineages [22]. Notably, this systemic endocrine vulnerability is not unique to traditional diabetogenic viruses; autopsy studies of fatal COVID-19 cases have similarly demonstrated widespread SARS-CoV-2 expression across the pancreas, pituitary, and thyroid glands [30].

The cellular kinetics driving this pancreatic damage have been partially clarified through in vitro human pancreatic organ cultures. These models demonstrate that viruses can establish both lytic and persistent infections within insulin-producing and non-insulin-producing endocrine cells. In lytic presentation, the primary cascade of cell death is characterized by early cellular pyknosis followed by delayed tissue necrosis, highlighting the direct cytopathic mechanisms by which viral pathogens compromise endocrine function. Indeed, viral infections—notably those driven by SARS-CoV-2—might be capable of contributing to the development of hyperglycemia [31,32].

## 3. SARS-CoV-2 Infection in Cats and Hyperglycemia

### 3.1. Natural Infection of SARS-CoV-2 in Cats

Following the emergence of COVID-19, natural SARS-CoV-2 infections were quickly identified in domestic cats [33]. Epidemiological and serological surveys conducted throughout the pandemic demonstrated high seroprevalence rates among pets living in close contact with COVID-19 patients [34]. However, the clinical presentation of virus infection in companion animals is highly variable. While exposure is common, classic respiratory or digestive symptoms are rarely reported [35]. A French cohort study showed that among four naturally infected cats, three presented as entirely asymptomatic, while one developed a mild coryza-like upper respiratory syndrome [36]. The virus isolates were confirmed as the Alpha variant [37].

As the pandemic evolved, the Alpha variant became widely distributed among companion animals globally, with documented cases spanning Texas (USA), Italy, and Spain [38,39,40,41]. Viral dynamics shifted with the emergence of subsequent lineages. Though the Delta variant was less frequently identified in Western pet populations, a well-documented natural case in Japan manifested with severe clinical disease characterized by acute respiratory distress, intractable coughing, sneezing, and purulent nasal discharge [42]. These findings indicate that SARS-CoV-2 variants might possess divergent pathogenic profiles in felids, raising questions regarding how shifting viral tropisms influence systemic and metabolic health in infected animals.

### 3.2. Experimental SARS-CoV-2 Infection and Feline Diabetes Induction, Viral Replication and Tissue Distribution

Experimental transmission studies utilizing ancestral human strains and environmental isolates have established divergent replication profiles between dogs and cats [41]. Following intranasal inoculation under BSL-4 conditions, infectious virions in cats were successfully recovered from the upper and lower respiratory tracts, as well as the salivary glands. While viral RNA was detected in the feline intestinal tract, no infectious virions were isolated from digestive tissues. Conversely, dogs demonstrated limited susceptibility; although viral RNA was transiently detected in the upper respiratory tract, no replication-competent virus was recovered, indicating restricted infectivity [41].

### 3.3. Metabolic Alterations and Pancreatic Pathology

To determine whether SARS-CoV-2 may alter glucose homeostasis in cats, Zhang and colleagues [19] monitored blood glucose kinetics post-infection. Seven days post-infection (dpi), inoculated cats exhibited hyperglycemia, while control animals remained normoglycemic. While post-mortem examinations revealed no macroscopic alterations in the pancreas, immunohistochemistry (IHC) demonstrated the expression of viral nucleocapsid (NP) and spike protein (SP) within both pancreatic ductal epithelial cells and endocrine cells in the islets of Langerhans (mainly glucagon-producing alpha cells).

These findings conflict with previously published results, but it is known that angiotensin-converting enzyme 2 (ACE2) expression varies across species and genotypes. Steenblock and colleagues [17] reported that 70% of COVID-19 cases expressed ACE2 in vascular cells, while the expression in beta cells of ACE2 was detected only in 30% of cases. In the cat pancreas, however, ACE2 expression was found mainly in glucagon-positive cells. Similar observations have been reported in humans. Autopsy studies by Tang and colleagues [43] detected SARS-CoV-2 antigens in pancreatic beta cells from individuals with COVID-19. Single-cell RNA sequencing and immunostaining confirmed that multiple types of pancreatic islet cells were affected by SARS-CoV-2, eliciting a cellular stress response with the production of chemokines. Reduced numbers of beta cells or reduced insulin production were found together with the enhanced expression of alpha cell and acinar cell markers, suggesting cellular trans-differentiation or dedifferentiation [44]. In cats, glucose tolerance tests performed in the early phase of infection may unmask the prediabetic stages as seen in the infection of mice with encephalomyocarditis virus (EMCV), Coxsackieviruses, and reovirus [45].

### 3.4. Alpha-Cell Tropism and Viral Receptors

Lineage-tracing and functional assays demonstrate that the pancreatic ductal epithelium acts as a progenitor pool for endocrine cells during stress or injury [14,39]. Pancreotropic viruses may capitalize on this plasticity [21,46], as surface receptors on ductal cells are likely conserved during differentiation into α-, β-, and δ-cells [47].

**Figure 1:**
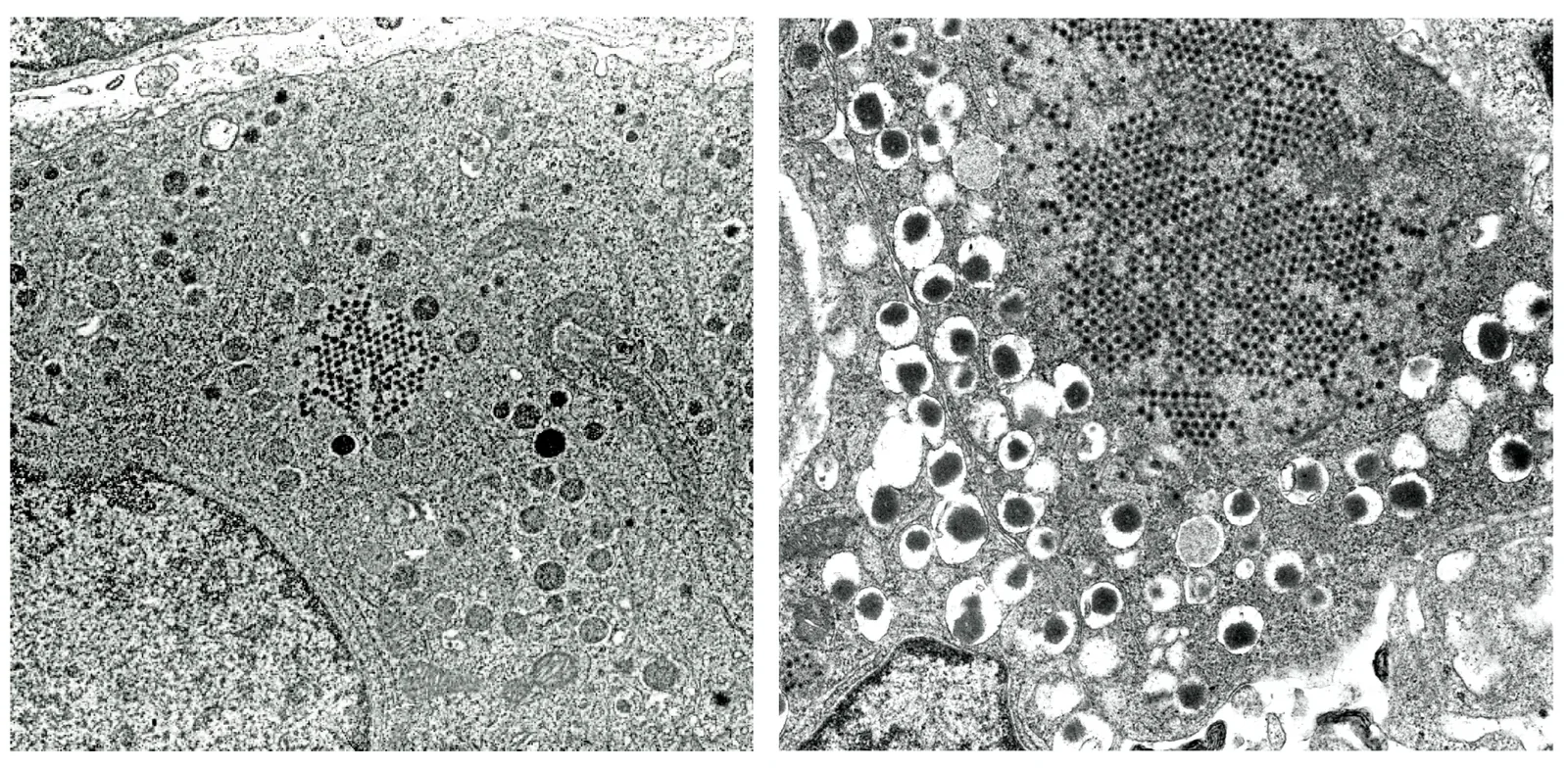
SJL mice 7 days after infection with reovirus type 1: transmission electron microscopy of pancreatic islets of Langerhans. Crystalline arrays of reovirus particles (70 nm) are visible in the cytoplasm of glucagon-producing alpha cells (**left**) and insulin-producing beta cells (**right**). The morphology of secretory vesicles differentiates alpha (electron-dense core apposing closely to the limiting membrane) and beta cells (core surrounded by a prominent electron-lucent halo) [48]. Courtesy of Springer, NY.

Using multi-spectral IHC, Zhang et al. mapped islet targets, revealing abundant viral NP co-expression with glucagon but minimal co-localization with insulin [19]—a selective tropism further confirmed using spike-protein antibodies. Simultaneous NP, glucagon, and ACE2 triple-staining identified peripheral alpha-cells as primary sites of ACE2-mediated entry. This peripheral distribution mirrors murine models of EMCV and reovirus type 1, where viral replication localizes to the alpha-cell-dense islet mantle [48]. Similarly, while reovirus type 3 initially targets the exocrine compartment [49], serial passage in islet cultures drives an adaptive tropism toward endocrine cells (Figure 1) [48], enriching for diabetogenic virions [8,29].

### 3.5. Comparative Feline Models of Infection with Variants of SARS-CoV-2: Metabolic Shift and Cardiorespiratory Reprogramming

To evaluate the impact of specific viral lineages, Rochowski and colleagues [20] inoculated intratracheally 24 specific-pathogen-free (SPF) domestic cats with a SARS-CoV-2 Delta variant isolate, evaluating pathology at 4 and 12 dpi. Infected cats exhibited significantly decreased serum insulin levels alongside elevated angiotensin II concentrations by 12 dpi. In spite of these systemic endocrine changes, viral RNA loads remained low within the pancreas and skeletal muscle. Instead, infection triggered localized metabolic adaptations, characterized by (a) increased protein expression of glucose transporters within pulmonary and cardiac tissues, and (b) upregulation of activated AMP-activated protein kinase (AMPK) levels in the heart. These findings suggest that SARS-CoV-2 drives a targeted metabolic reprogramming of the cardiorespiratory axis to satisfy the bioenergetic demands of viral replication [20]. This model establishes a framework for evaluating metabolic interventions during viral pathogenesis. For instance, the classic antidiabetic agent metformin counteracts these alterations by inhibiting mitochondrial Complex I, thereby elevating the AMP/ATP ratio to activate the AMPK pathway. Notably, histopathological evaluation showed no widespread, significant reduction in beta-cell numbers, though focal lymphocytic infiltration and inflammatory cytokine expression were detected in pancreatic islets of a single animal at 12 dpi [20].

### 3.6. Transmission Dynamics and Tissue Tropism

Characterizing broader infection kinetics, Nooruzzaman and Diel [50] confirmed high susceptibility and efficient horizontal virus transmission in domestic cats. Consistent with human COVID-19 pathology, clinical infectivity and histopathological lesions were concentrated within the respiratory tract. Infected animals generated robust, multi-faceted immune profiles (innate, cell-mediated, and humoral), allowing rapid viral clearance. Transmission to contact animals occurred via oral and nasal secretions as early as 1–2 dpi, with peak shedding at day 7 post-contact [50]. Conversely, separate evaluations highlighted the lack of structural lesions or viral antigen localization in adjacent tissues, including the palate, tonsils, retropharyngeal lymph nodes, and myocardium [50].

### 3.7. Summary of Divergent Evidence in Feline Pancreatic Pathology

The current literature presents distinct phenotypic variations regarding how SARS-CoV-2 impacts the feline pancreas and glucose homeostasis. Table 2 summarizes the main differences among the principal studies.

These divergent phenotypes—spanning from acute islet infiltration and hyperglycemia to isolated respiratory pathology with preserved beta-cell mass—likely arise from variations in inoculum dosage, viral strain, or host demographics, all of which are dissected comprehensively in the Discussion.

## 4. Main Antiviral Agents for Treating SARS-CoV-2 and Other Coronavirus Infections

This section reviews the main antiviral agents for treating coronavirus-related clinical manifestations in humans and animals (Table 3).

RdRp inhibitors disrupt viral genome replication: remdesivir causes premature and delayed chain termination, while molnupiravir induces lethal mutagenesis in the nascent viral RNA. Protease inhibitors block the polyprotein cleavage required for viral assembly. While ensitrelvir and bofutrelvir directly target the main protease (3CLpro/Mpro), ritonavir—a weak HIV protease inhibitor—is primarily used to inhibit CYP3A4 and prolong the half-life of co-administered therapeutics. The regulatory status of these anti-coronavirus drugs spans human and veterinary medicine (Table 4), with notable variations across the US (FDA), Europe (EMA), and Japan (PMDA).

In swine, experimental studies document the efficacy of Remdesivir, Molnupiravir, and various 3CLpro inhibitors against porcine coronaviruses (PEDV, PDCoV, TGEV, and SADS-CoV) [59]. Lacking formal regulatory approvals for animal-specific coronavirus antivirals, veterinary practice relies on compounding frameworks or the use of off-label human medications [51]. Three primary agents are utilized: (a) GS-441524: The oral parent nucleoside of remdesivir (sourced via special import or compounding); (b) Remdesivir: Used off-label as an injectable drug to stabilize critically ill cats before transitioning to oral GS-441524, as both yield the same active triphosphate metabolite in feline cells; and (c) Molnupiravir: Deployed off-label as a second-line therapy in cases of clinical resistance to GS-441524 or FIP/SARS-CoV-2 co-infections.

The broad-spectrum efficacy of RdRp and main protease (Mpro) inhibitors across the Coronaviridae underscores their value in pandemic preparedness and veterinary therapeutics [56,60].

In conclusion, combination regimens targeting two distinct enzymes (e.g., RdRp and Mpro) accelerate viral clearance and may reduce the frequency of long-COVID syndrome. Currently recommended for selected human patients [61] and feline FIP [62], dual-inhibitor therapy holds promise for mitigating the cardiologic, endocrine, and metabolic sequelae of coronavirus infections across species.

## 5. Discussion

### 5.1. The Bidirectional Relationship Between Coronaviruses and Glycemic Control

Metwally et al. [63] highlighted that pre-existing diabetes constitutes a critical risk factor for severe clinical forms of COVID-19, with affected patients exhibiting significantly higher mortality rates. Concurrently, they suggested that the virus itself may directly precipitate new-onset metabolic dysfunction [64]. Over time, this bi-directional intersection between SARS-CoV-2 and glucose homeostasis has become more evident. Epidemiological data in humans indicate that SARS-CoV-2 infection may contribute to new-onset diabetes, manifesting as acute hyperglycemia in patients with no prior history of altered glycemic control, or as severe diabetic ketoacidosis (DKA) in patients with pre-existing disease [1].

Mechanistically, the SARS-CoV-2 spike protein interacts with the renin–angiotensin–aldosterone system (RAAS), gaining cellular entry through binding to the ACE2 receptor. Given that these receptors are expressed across both the exocrine and endocrine compartments of the pancreas, viral entry into pancreatic beta-cells—coupled with local ACE2 downregulation and the subsequent accumulation of angiotensin II—is increasingly recognized as a primary driver of this diabetogenic effect [65]. Consequently, the rising incidence of post-acute metabolic complications necessitates glycemic management during both acute COVID-19 and the chronic course of long-COVID.

### 5.2. Pancreatic ACE2 Expression and Comparative Viral Tropisms

The precise spatial topography of human ACE2 expression within the pancreas remains a critical area of investigation. While early, low-resolution tissue profiling suggested widespread, non-specific pancreatic expression [66], recent high-resolution spatial analyses confirm that ACE2 is highly localized within both α- and β-cells of humans [67]. This distinct endocrine tropism closely mirrors the pancreatic affinity observed in other classical diabetogenic viruses; for instance, enteroviruses [68,69,70], including group B coxsackieviruses [71] and rotaviruses [72], have long been implicated in the autoimmune and cytopathic pathogenesis of type 1 diabetes. Consequently, longitudinal metabolic monitoring of post-COVID-19 human patients is paramount to deciphering how acute viral insults drive long-term progression toward diverse diabetic phenotypes.

### 5.3. Methodological Challenges and Discrepancies in Autopsy Data

Reports mapping the specific cellular subsets harboring SARS-CoV-2 antigens or RNA within the pancreas have yielded conflicting results in both human and feline cohorts. Human post-mortem series have variously demonstrated that viral proteins and mRNA localize strictly to insulin-positive beta-cells and adjacent islet architectures [73], or reside exclusively within the exocrine ductal epithelium while sparing the endocrine tissue [74]. Conversely, a third subset of literature indicates that both the endocrine and exocrine compartments are concurrently affected [17,43,75]. These divergent histopathological findings likely stem from inter-individual heterogeneity in the baseline expression and spatial dynamics of viral receptors and entry co-receptors. Furthermore, technical variations in co-staining methodologies used to evaluate pancreatic hormones alongside viral antigens or RNA play a significant confounding role. For human autopsy tissue, the post-mortem interval (PMI) is an additional critical variable. Indeed, rapid post-mortem pancreatic autolysis can swiftly degrade viral components, leading to false-negative staining.

Additionally, since SARS-CoV-2 infection may induce beta-cell dedifferentiation or profound degranulation, infected cells frequently exhibit drastically reduced insulin content. Consequently, cells may stain negative for insulin despite retaining their beta-cell lineage. Incorporating structural, non-hormonal beta-cell markers—such as the transcription factor NK6 homeobox 1 (NKX6.1) [76]—will be essential in future studies to accurately map viral distribution and quantify true beta-cell mass changes post-infection.

### 5.4. A Framework for Investigating Coronavirus-Induced Hyperglycemia

Building on historical evidence from Coxsackievirus B4-associated diabetic ketoacidosis and hyperglycemia [2], we propose a four-component translational framework to systematically investigate SARS-CoV-2-mediated pancreatic dysfunction:

**Model Development:** Establish and validate an animal model optimized to assess the longitudinal diabetogenic potential of SARS-CoV-2 infection.

**Host Selection:** Employ highly susceptible feline strains or develop transgenic models expressing human ACE2 under pancreas-specific promoters to faithfully reproduce natural infection dynamics. In the EMC or Coxsackievirus infection mouse model, the inbred strains fell into three categories: 1. strains that became hyperglycemic and had abnormal glucose tolerance tests; 2. strains that only showed abnormal glucose tolerance tests; and 3. strains that showed neither abnormality [2].

**Viral Characterization:** Isolate and sequence potentially diabetogenic SARS-CoV-2 strains using plaque assays to identify genomic variants associated with enhanced beta-cell tropism. All work needs to be conducted in accordance with Dual-Use Research of Concern (DURC) protocols and institutional biosafety guidelines.

**Spatiotemporal Mapping:** Apply high-resolution immunofluorescence and multiplex immunohistochemistry to localize viral infection and antigen expression within the pancreatic islet microarchitecture.

### 5.5. Environmental Triggers of Endocrine Failure

Clinical diabetes requires both genetic predisposition and environmental triggers; animal models and twin studies indicate that viral infections represent the primary environmental risk factor [77]. Common respiratory and gastrointestinal viruses promote systemic infection and hyperglycemia through three interconnected mechanisms: direct beta-cell dysfunction and apoptosis, local inflammatory cytokine production, and downstream T-cell-mediated autoimmunity [78]. Clarifying how SARS-CoV-2 triggers acute and chronic pancreatic endocrine dysfunction is essential for predicting, treating, and preventing diabetes in humans and other susceptible species, or susceptible animal strains.

## 6. Conclusions

This review establishes a mechanistic link between coronavirus infections and pancreatic pathology, integrating clinical evidence from human COVID-19 with feline models of alpha- and beta-coronavirus infection. The evidence demonstrates that coronaviruses compromise pancreatic integrity through three primary pathways: direct cellular tropism, receptor-mediated RAAS dysregulation, and inflammatory cascade activation.

Our proposed model of the ductal-to-endocrine network provides a mechanistic explanation for how pancreotropic viruses may disseminate from the exocrine epithelium into endocrine islets in the course of infection. Integration of human histopathological data with validated feline models will strengthen our ability to assess metabolic consequences of coronavirus infections and identify individual factors of susceptibility to virus-associated hyperglycemia.

## Figures and Tables

**Table 1:** Viruses causing hyperglycemia in animals.

Animal Host	Virus	Viral Family/Genus	Primary Mechanism/Pathogenesis	Metabolic & Clinical Outcome	Key References
Bovines (Cattle)	Foot-and-Mouth Disease Virus (FMDV)	Picornaviridae, Aphtovirus	Lytic infection of pancreatic islet beta-cells leading to loss of insulin synthesis.	Transient or permanent hyperglycemia, chronic insulin deficiency.	Pedini 1962 [5]
Mice	Encephalomyocarditis Virus, Variant D (EMC-D)	Picornaviridae, Cardiovirus	Cytolytic destruction of pancreatic beta-cells without autoimmune T-cell involvement.	Type 1-like insulin-dependent diabetes mellitus (rapid onset within 3–5 days).	Craighead 1968 [6]
Mice	Coxsackievirus B	Picornaviridae, Enterovirus	Beta-cell cytolysis and with virus-induced autoimmunity (bystander activation and release of sequestered autoantigens).	Acute or chronic hyperglycemia, autoimmune Type 1-like diabetes susceptible strains (e.g., SJL/J, CD-1).	Horwitz 1998 [7]
Mice	Reovirus type 1 and 3	Reoviridae, Orthoreovirus	Infection of alpha, beta, delta pancreatic islet cells with autoantibodies against islet cells and other endocrine organs.	Moderate hyperglycemia and polyendocrine autoimmunity.	Onodera 1978 [8]
Mice	Murine Cytomegalovirus (MCMV)	Herpesviridae, Betaherpesvirinae	Infection of pancreatic acinar and stromal cells with focal pancreatitis, immune infiltration, cytokine-mediated beta-cell dysfunction.	Non immune-mediated glucose intolerance.	Hayashi 1985 [9]
Mice	Lymphocytic Choriomeningitis Virus (LCMV)	Arenaviridae, Mammarenavirus	Virus-triggered autoimmune beta-cell destruction mediated by CD8+ T lymphocytes targeting viral antigens in islet cells.	Rapid-onset autoimmune type 1-like diabetes with hyperglycemia and insulitis.	Oldstone 1991 [10]
Monkeys (Rhesus, Cynomolgus)	Coxsackievirus B4	Picornaviridae, Enterovirus	Viral insulitis, focal necrosis of islet beta-cells, inflammatory depression of insulin secretion.	Impaired glucose tolerance, transient or overt hyperglycemia.	Yoon 1986 [11]

**Table 2:** Metabolic and pathological changes in cats infected with SARS-CoV-2.

Study	Primary Pancreatic/Metabolic Findings	Islet Architecture Status
Zhang et al. [19]	Marked hyperglycemia; strong viral NP expression localized to islet alpha-cells.	Documented IHC alterations.
Rochowski et al. [20]	Significantly decreased serum insulin; cardiac/pulmonary metabolic reprogramming.	Preserved beta-cell numbers; minimal islet inflammation (1/24 cats).
Nooruzzaman & Diel [50]	Robust respiratory replication and shedding; clear systemic immune response.	No primary endocrine or pancreatic deficits reported.

**Table 3:** Comparison of anti-coronavirus drugs based on their primary target, mechanism of action, and pharmacology.

Antiviral Drug	Primary Target	Mechanism of Action	Pharmacology	References
**Remdesivir**	Inhibitor of viral RdRp	Prodrug of GS-441524 (adenosine analog). Converted into active nucleoside triphosphate, it competes with natural ATP for incorporation into the nascent RNA strand, causing premature and delayed chain termination.	Broad-spectrum efficacy due to highly conserved RdRp target across *Coronaviridae*.	Brüssow 2026 [51]
				Tasker 2026 [52]
**Molnupiravir**	Inhibitor of viral RdRp	Metabolized into beta-D-hydroxycytidine triphosphate, incorporated into viral RNA. During replication cycles, the viral polymerase misinterprets the embedded modified nucleotides. This results in accumulating transition mutations that make the viral genome non-functional.	Estimated 2–10 times higher potency than remdesivir in certain in vitro models; requires monitoring for mutagenic risks.	Khalil 2026 [53]
**Ritonavir**	Inhibitor of main viral protease	Targets the viral 3C-like protease (3CLpro)/aspartic protease. However, as monotherapy, its direct benefit against Coronaviruses is negligible.	Primary function: by inhibiting host CYP3A4 enzymes extends the half-life of co-administered drugs.	Loos 2023 [54]
**Ensitrelvir**	Inhibitor of main viral protease	Non-covalent, non-peptide inhibitor that binds to 3CLpro, halting viral polyprotein processing and protease-derived cellular damage.	High affinity for wild-type protease with an IC_50_ = 0.049 mM, comparable to nirmatrelvir.	Syed 2024 [55]
				Mik 2026 [56]
**Bofutrelvir**	Inhibitor of main viral protease	Inhibitor of the main protease 3CLpro, blocking polyprotein cleavage and reducing viral yield and RNA copy numbers.	IC_50_ = 0.053 μM, TR-FRET assays. Scientific profiles emerging in 2025–2026.	Wang 2024 [57]
				Ye 2026 [58]

**Table 4:** Approval status of antiviral drugs for treating SARS-CoV-2 infection in humans.

Antiviral Drug	Primary Mechanism	United States (FDA)	Europe (EMA)	Japan (PMDA)
**Remdesivir**	RdRp Inhibitor (chain termination)	**Fully Approved**	**Fully Approved**	**Fully Approved**
**Nirmatrelvir/Ritonavir**	3CLpro inhibitor plus CYP3A4 booster	**Fully Approved**	**Fully Approved**	**Fully Approved**
**Molnupiravir**	RdRp Inhibitor (lethal mutagenesis)	**Emergency Use Authorization**	Not Approved (refused by EMA in 2023)	**Fully Approved**
**Ensitrelvir**	3CLpro Inhibitor (non-covalent)	Not Approved (Phase 3 Trials)	Not Approved (Phase 3 Trials)	**Fully Approved** (Conditional/Emergency)

## Data Availability

No new data were created or analyzed in this study. Data sharing is not applicable to this article.

## Abbreviations

ACE2	Angiotensin-converting enzyme 2
AMPK	AMP-activated protein kinase
3CLpro	3C-like protease
CYP3A4	Cytochrome P450 3A4
DKA	Diabetic ketoacidosis
EMCV	Encephalomyocarditis virus
FECV	Feline enteric coronavirus
FCoV	Feline coronavirus
FIP	Feline infectious peritonitis
FIPV	Feline infectious peritonitis virus
IC_50_	Half-maximal inhibitory concentration
IHC	Immunohistochemical
Mpro	Main protease enzyme
NP	Nucleocapsid protein
RAAS	Renin–angiotensin–aldosterone system
RdRp	RNA-dependent RNA-polymerase
SP	Spike protein
T1DM	Type 1 diabetes mellitus
TR-FRET	Time-resolved fluorescence resonance energy transfer
